# Draft Genome Sequence of Listeria monocytogenes CIIMS-NV-3, a Strain Isolated from Vaginal Discharge of a Woman from Central India

**DOI:** 10.1128/MRA.01553-18

**Published:** 2019-02-07

**Authors:** Pooja M. Kishnani, Nitin V. Kurkure, Sukhadeo B. Barbuddhe, Swapnil P. Doijad, Trinad Chakraborty, H. F. Daginawala, Lokendra K. Singh, Rajpal S. Kashyap

**Affiliations:** a Research Laboratory, Central India Institute of Medical Sciences (CIIMS), Bajaj Nagar, Nagpur, India; b Nagpur Veterinary College, Nagpur, India; c ICAR–National Research Centre on Meat, Hyderabad, India; d Institute of Medical Microbiology, Biomedizinisches Forschungszentrum, Giessen, Germany; Georgia Institute of Technology

## Abstract

We present here the draft genome sequence of Listeria monocytogenes CIIMS-NV-3, a serovar 4b strain isolated from the vaginal swab of a female patient from central India. The availability of this genome may provide useful information on virulence characteristics for comparative genomic analysis.

## ANNOUNCEMENT

Listeria monocytogenes is a Gram-positive bacterium responsible for listeriosis in humans. Globally, more than 90% of cases of listeriosis are caused by serovar 1/2a, 1/2b, or 4b (1). Clinical manifestations of an invasive form of infection are meningoencephalitis, meningitis, and septicemia (2). This pathogen can be transmitted via vertical transmission from mothers to neonates during passage through the birth canal. The probability of occurrence of neonatal infection may increase with a biofilm-producing clinical isolate (3). Here, we present the draft genome sequence of L. monocytogenes CIIMS*-*NV-3, isolated from the vaginal discharge of a female with a history of gynecological problems hospitalized in the Government Medical College in Nagpur, India.

For isolation of the *Listeria* isolate, the vaginal swab was collected in a sterile vial and stored at 4°C until further analysis. The sample was processed according to the method of the U.S. Department of Agriculture (USDA). Brieﬂy, a swab was directly inoculated into 10 ml of University of Vermont medium 1 (UVM 1) and incubated overnight at 30°C. The enriched UVM 1 inoculum (0.1 ml) was then transferred to UVM 2 and again incubated overnight at 30°C. The inoculum from enriched UVM 2 was streaked on PALCAM agar (HiMedia Laboratories, Mumbai, India). The inoculated plates were incubated at 37°C for 48 h. The presumed *Listeria* colonies were further characterized morphologically and biochemically. Typical colonies were veriﬁed with Gram staining, a catalase reaction, an oxidase test, a tumbling motility test at 20°C to 25°C, methyl red-Voges-Proskauer reactions, a CAMP test with Staphylococcus aureus, nitrate reduction, fermentation of sugars (rhamnose, xylose, mannitol, and methyl-α-d-mannopyranoside), and hemolysis on 5% sheep blood agar. The isolate was tested for its pathogenicity with a phosphatidylinositol-speciﬁc phospholipase C (PI-PLC) assay.

The genomic DNA was isolated using a Qiagen genomic DNA extraction kit from an 18-h culture on brain heart infusion (BHI) agar. Libraries were constructed using a paired-end library (2 × 250 bp) using a v2 chemistry reagent kit. The sequencing of the isolate was performed on an Illumina HiSeq 2500 platform. The reads were trimmed with Trimmomatic v0.36 (4). *De novo* assembly was performed with SPAdes v3.11.1 (5).

The sequencing showed 5,826,618 reads (250× read coverage). The draft genome sequence of CIIMS-NV-3 resulted in nine contigs with 521× assembly coverage and a total assembly size of 2,954,075 bp, with a GC content of 37.88%. CIIMS-NV-3 showed >95% average nucleotide identity with BLAST (ANIb) to L. monocytogenes NCTC 10357^T^ (accession number NZ_LT906436), which indicates a *sensu stricto* isolate of L. monocytogenes. The genome was annotated with PROKKA v1.13 and shown to possess 3,035 genes, with 2,942 as coding sequences. Further genome analysis for virulence genes was done with BLASTp (95% coverage and 95% identity) against the virulence factor database (VFDB). CIIMS-NV-3 encoded *Listeria* pathogenic island I (LIPI-I) and LIPI-III. Other virulence genes, such as intact *inlA*, *inlB*, *inlC*, *inlF*, *inlJ*, *bsh*, *ami*, *agrA*, *agrC*, *srtA*, *srtB*, *virR*, *virS*, and *fpbA*, were also noted. Complete flagellar and chemotaxis gene clusters of were also observed (6).

For characterization of the *Listeria* isolate, a multilocus sequence typing (MLST) scheme was used. In MLST, each species is characterized by a series of integers, which correspond to the alleles at the housekeeping loci. MLST was carried out with the FASTA sequence on the multilocus sequence typing (MLST) server provided by the Center of Genomic Epidemiology (https://cge.cbs.dtu.dk/services/MLST/). *In silico* MLST identified the isolate as belonging to sequence type 328 (ST-328, clonal complex 1, lineage I), which had been reported to be the predominant sequence type in India (7). All bioinformatics analysis was carried out with the default settings.

The genome will be further studied to determine the characteristics of the strain for comparative analysis. In developing countries such as India, the incidence of L. monocytogenes from clinical cases is underreported due to the lack of awareness and proper diagnostic assays. Nonetheless, public accessibility of the genomes of L. monocytogenes isolates, such as that from the vaginal discharge of a female patient, is important from the clinical and epidemiological points of view (8, 9).

### Data availability.

The annotated whole-genome sequence of this strain has been deposited in GenBank under accession number CP031674. The SRA accession number for the raw reads is SRR8383222.
